# Clinico-Hematological and Immunophenotypic Profile of Leukemia Patients at a Tertiary Care Center in Jharkhand, India: A Cross-Sectional Study

**DOI:** 10.7759/cureus.107063

**Published:** 2026-04-14

**Authors:** Abhirup Shome, Manoj K Paswan, Anshu Jamaiyar, Abhimanyu Shome

**Affiliations:** 1 Pathology, Rajendra Institute of Medical Sciences, Ranchi, IND; 2 Internal Medicine, Rajendra Institute of Medical Sciences, Ranchi, IND

**Keywords:** clinico-hematological profile, flow cytometry, immunophenotyping, jharkhand, leukemia

## Abstract

Background

Leukemia represents a significant and rising health burden in India. Despite this, a government-maintained population-based cancer registry is lacking in Jharkhand. This study aimed to characterizethe clinico-hematological and immunophenotypic profile of newly diagnosed leukemia cases from a tertiary care center in this underrepresented region.

Methods

This was a hospital-based, cross-sectional study conducted in the Department of Pathology, Rajendra Institute of Medical Sciences, Ranchi, India, over a two-year period (February 2024 to January 2026). A total of 151 newly diagnosed leukemia cases were evaluated. Peripheral blood smear, bone marrow aspiration, and immunophenotyping by flow cytometry were performed in accordance with the World Health Organization Classification of haematolymphoid tumours, Fifth Edition (WHO-HAEM5) and International Consensus Classification (ICC) criteria. Data were recorded in Microsoft Excel (Redmond, USA) and analyzed using Jamovi v2.7.17.

Results

Of the 151 patients, the mean age was 36.7 ± 19.8 years, with a male predominance (74.2%; M:F = 2.9:1). The majority of patients were from a rural background (79.5%). Chronic myeloid leukemia, chronic phase (CML-CP) was the most frequent diagnosis (27.8%), followed by acute myeloid leukemia (AML) and B-cell acute lymphoblastic leukemia (B-ALL) (24.5% each), chronic lymphocytic leukemia (CLL) (12.6%), T-cell acute lymphoblastic leukemia (T-ALL) (8.6%), and mixed phenotype acute leukemia (MPAL) (2.0%). Fever and bleeding manifestations were more prevalent in acute leukemias (p < 0.001). Severe thrombocytopenia was almost universal in acute leukemias. CD34-positive AML showed higher WBC counts, higher blast percentages, and lower hemoglobin and platelet counts compared to CD34-negative cases (all p < 0.05). CD10-negative B-ALL showed higher WBC counts (p < 0.001), higher blast percentages (p = 0.008), and lower platelet counts (p = 0.048) compared to CD10-positive cases; these findings should be regarded as exploratory given the small subgroup size (n = 5).

Conclusions

This study establishes a baseline clinico-hematological and immunophenotypic profile of leukemias in Jharkhand. These findings reflect the need for a state-level population-based cancer registry and strengthened diagnostic infrastructure in the region.

## Introduction

Leukemia represents a heterogeneous group of blood disorders, characterized by malignant transformation of the hematopoietic stem cells, leading to replacement of the normal bone marrow cells by neoplastic blast cells, with or without spillage into the peripheral blood [1]. Globally, leukemia represents a significant health burden for cancer incidence and mortality, with an estimated 400,000 new cases diagnosed annually worldwide [2,3]. Various population-based registries from Western nations report higher incidence in older adults with a predilection for the chronic variants; however, data from South and Southeast Asia report higher incidence in younger populations and a higher proportion of acute leukemia at presentation [3-5].

In India, leukemias are one of the leading causes of hematological malignancies, and recent reports suggest that the incidence is rising at a disproportionately rapid rate compared to global data [3-5]. Despite this, many Indian states lack data on various cancers, including hematological cancers [5]. Additionally, some states, including Jharkhand, lack a government-maintained cancer registry, thereby creating a significant gap in regional epidemiological understanding [6].

Historically, the diagnosis of leukemia was dependent on morphology followed by cytochemistry. However, with the advent of modern diagnostic techniques, the incorporation of immunophenotyping by flow cytometry, cytogenetics, and molecular profiling has become the standard recommendation as per WHO-HAEM5 and the ICC [7-10]. Previous studies from tertiary care centers in India highlight the feasibility of immunophenotyping by flow cytometry in various tertiary care centers in the country [11,12].

Despite the lack of data, no comprehensive clinico-hematological study comprising the full spectrum of leukemia subtypes has been conducted in the region. The present study was conducted with the aim of characterizing the demographic, clinical, hematological, and immunophenotypic profile of newly diagnosed leukemia cases at a tertiary care center in Jharkhand, in a hospital-based cross-sectional design. The objective was to establish a preliminary baseline dataset from this underrepresented region, which may serve as a reference for future population-based studies and contribute to the evidence base for regional healthcare planning.

## Materials and methods

Study design, setting, and duration

This was a hospital-based, cross-sectional study conducted over a period of two years, from February 2024 to January 2026, at the Department of Pathology, Rajendra Institute of Medical Sciences (RIMS), Ranchi, Jharkhand, India.

Ethics statement

This study was approved by the Institutional Ethics Committee of RIMS, Ranchi, India (IEC Registration No. ECR/769/INST/JH/2015/RR-21; Letter No. 85, Dated: 01/02/2025). Informed consent was obtained from adult patients, and for the pediatric population, consent was obtained from their parents or legal guardians. This study was conducted in accordance with the Declaration of Helsinki. Patient confidentiality was maintained throughout the study.

Study population and eligibility criteria

All patients of any age group presenting with clinical features and hematological findings suggestive of leukemia, confirmed on peripheral blood smear (PBS) or bone marrow aspiration (BMA), were included in the study. Patients already diagnosed with any type of malignancy or receiving treatment for leukemia or other types of cancer, and those denying consent were excluded from the study.

Sample size

Due to the lack of a population-based cancer registry, exact prevalence data were unavailable; hence, 50% was assumed to ensure the maximum possible sample size estimate [6]. Using the Cochrane formula, with a 95% confidence interval (Z = 1.96), an assumed prevalence of 0.5, and a margin of error of 8%, the calculated sample size was 151. A margin of error of 8% was chosen instead of the conventional 5% due to the relative rarity of hematological malignancies and the practical limitations of a single-center study.

Peripheral blood smear

Venous blood was collected in EDTA-anticoagulated tubes and processed using an automated hematology analyzer (Horiba Yumizen H2500; Horiba, Ltd., Kyoto, Japan). Hemoglobin (g/dL), total leukocyte count (TLC) (/mm³), and platelet count (/mm³) were recorded for all patients. PBS was prepared and stained with Leishman-Giemsa (LG) stain and examined under a microscope for blast count and differential count.

Bone marrow aspiration (BMA)

BMA was performed in all patients with suspicion of leukemia. The posterior superior iliac spine was the preferred site. The anterior superior iliac spine was selected in obese patients, the sternum in cases of dry tap from the iliac crest, and the tibia for children younger than two years of age. A 14-gauge (G) Salah's needle was used in adults, 16G for children aged two to ten years, and 18G for those under the age of two years. The smears were stained with LG stain and examined by consultant hematopathologists in correlation with PBS and clinical features.

Flow cytometry and immunophenotyping

Multicolor flow cytometry was performed on all cases of acute leukemia (AML, B-ALL, T-ALL, and MPAL) and CLL. Bone marrow aspirate was the preferred sample; however, peripheral blood was used in patients with a TLC above 30,000/mm³. Samples were processed in accordance with the standard operating protocol of BD Biosciences on a BD FACSCanto II flow cytometer (BD Biosciences, San Jose, CA, USA) [13]. Data acquisition and automated compensation were performed using BD FACSDiva software. Compensated flow cytometry standard (FCS) files were subsequently exported to Floreada (Dotmatics, Bristol, UK), a web-based flow cytometry analysis platform, for gating and marker quantification [26].

Instrument setup utilized an unstained control tube per case to establish baseline settings. Automated compensation was calculated using single-stained controls within BD FACSDiva software prior to acquisition. Data acquisition was performed at a low flow rate, with a standardized stopping gate of 50,000 events per tube. A sequential gating strategy was employed: doublet discrimination was first performed via FSC-A versus FSC-H to isolate singlets, followed by morphological viability gating via FSC versus SSC to exclude debris and non-viable cells, and finally primary analytical gating on a CD45 versus SSC plot, with blast isolation in the CD45 dim-to-moderate, low-SSC region. A separate lymphocyte gate defined by bright CD45 expression and low SSC was used for CLL analysis and as an internal quality control. Lineage assignment positivity cutoffs were defined as ≥20% for surface antigens and ≥10% for cytoplasmic markers. Instrument quality control was performed periodically by a specialized departmental technician using standardized calibration beads.

A standardized antibody panel was used across all cases of acute leukemia and CLL. Myeloid markers included CD13, CD33, CD117, HLA-DR, and cytoplasmic myeloperoxidase (cMPO); B-lineage markers included CD19, CD10, CD79a, CD20, and CD23; T-lineage markers included cytoplasmic CD3 (cCD3), CD7, and CD5; maturity markers included CD34, terminal deoxynucleotidyl transferase (TdT), and CD38; and CD200 was included for CLL assessment. For acute leukemia cases, the panel was organized into four tubes: the B-tube comprised CD20 (FITC), CD10 (PE), CD34 (APC), CD19 (PE-Cy7), CD38 (PerCP-Cy5.5), and CD45 (APC-Cy7); the T-tube comprised CD8 (FITC), CD5 (PE), CD7 (APC), CD4 (PE-Cy7), CD3 (PerCP-Cy5.5), and CD45 (APC-Cy7); the myeloid tube comprised CD64 (FITC), CD33 (PE), CD117 (APC), CD13 (PE-Cy7), HLA-DR (PerCP-Cy5.5), and CD45 (APC-Cy7); and the cytoplasmic tube comprised cMPO (FITC), cytoplasmic CD79a (PE), CD34 (APC), cytoplasmic CD3 (PerCP-Cy5.5), and CD45 (APC-Cy7). For CLL cases, a single tube comprised CD23 (FITC), CD200 (PE), CD5 (PerCP-Cy5.5), CD20 (APC), CD19 (PE-Cy7), and CD45 (APC-Cy7). This was done in accordance with ICMR guidelines and the Bethesda International Consensus recommendations [14,15].

Diagnostic criteria

The final diagnosis was established by integrating morphological and immunophenotypic findings in accordance with WHO-HAEM5 and ICC [7-10]. Cytogenetic and molecular studies were not performed due to resource constraints. For chronic myeloid leukemia (CML), the diagnosis was confirmed by detection of the BCR-ABL1 fusion transcript on qualitative reverse transcription polymerase chain reaction (RT-PCR), performed by the Department of Genetics at RIMS, Ranchi. Phase assignment was based on blast percentage [7].

Statistical analysis

Data were recorded in Microsoft Excel and analyzed using Jamovi v2.7.17 (The Jamovi Project, Sydney, Australia). Descriptive analyses, including frequencies, proportions, mean ± standard deviation (SD), and medians, were calculated for variables across disease subgroups. Categorical variables were compared using the Chi-square test or Fisher's exact test for variables with an expected cell frequency of five or fewer. Continuous variables were compared using appropriate parametric or nonparametric tests based on the distribution of data. For multi-group comparisons, normally distributed variables were analyzed using Welch's one-way ANOVA, while variables with evidence of skewness (WBC count and platelet count) were analyzed using the Kruskal-Wallis test. Two-group comparisons were performed using Welch's independent samples t-test (unequal variances assumed). For subgroup comparisons involving very small sample sizes (n < 10), the Mann-Whitney U test was used as a nonparametric alternative.

## Results

A total of 151 newly diagnosed leukemia patients were included in this study. The mean age was 36.7 ± 19.8 years (range: 3-75 years). The largest age group comprised adults aged 30-44 years (n = 38; 25.2%), followed by the middle age group 45-59 years (n = 34; 22.5%). A clear male predominance was observed (n = 112; 74.2%) with a male-to-female ratio of 2.9:1. The majority of patients were from a rural background (n = 120; 79.5%), and farmers were the largest occupational group (n = 50; 33.1%), closely followed by students (n = 43; 28.5%) and laborers (n = 30; 19.9%).

CML-chronic phase (CML-CP) was the most frequent diagnosis (n = 42; 27.8%), followed by AML and B-ALL (n = 37 each; 24.5%), CLL (n = 19; 12.6%), T-ALL (n = 13; 8.6%), and MPAL (n = 3; 2.0%). All 42 CML-CP cases were confirmed BCR-ABL1 positive by qualitative RT-PCR. The demographic profile and disease distribution are summarized in Table 1.

**Table 1:** Demographic profile and disease distribution of patients with hematological malignancies (n = 151) CML-CP: chronic myeloid leukemia, chronic phase; B-ALL: B-cell acute lymphoblastic leukemia; AML: acute myeloid leukemia; CLL: chronic lymphocytic leukemia; T-ALL: T-cell acute lymphoblastic leukemia; MPAL: mixed phenotype acute leukemia. † Others include business owners, drivers, homemakers, security personnel, service sector employees, and skilled artisans.

Variables	Categories	Number of cases; n (%)
Age group	Pediatric (≤14 years)	27 (17.9%)
	Adolescent (15–29 years)	30 (19.9%)
	Adult (30–44 years)	38 (25.2%)
	Middle-aged (45–59 years)	34 (22.5%)
	Elderly (≥60 years)	22 (14.6%)
Sex	Male	112 (74.2%)
	Female	39 (25.8%)
Residence	Rural	120 (79.5%)
	Urban	31 (20.5%)
Occupation	Farmer	50 (33.1%)
	Student	43 (28.5%)
	Laborer	30 (19.9%)
	Retired	7 (4.6%)
	Housewife	6 (4.0%)
	Others†	15 (9.9%)
Disease distribution	CML-CP	42 (27.8%)
	B-ALL	37 (24.5%)
	AML	37 (24.5%)
	CLL	19 (12.6%)
	T-ALL	13 (8.6%)
	MPAL	3 (2.0%)

Significant inter-group differences were observed for fever, bleeding manifestations, lymphadenopathy, splenomegaly, hemoglobin levels, TLC, platelet count, and bone marrow blast percentage (all p < 0.001). However, hepatomegaly and sternal tenderness did not reach statistical significance (p = 0.326 and p = 0.067, respectively). Clinical features across subtypes are summarized in Table 2.

**Table 2:** Clinical features across hematological malignancy subtypes (n = 151) AML: acute myeloid leukemia; B-ALL: B-cell acute lymphoblastic leukemia; T-ALL: T-cell acute lymphoblastic leukemia; CML-CP: chronic myeloid leukemia, chronic phase; CLL: chronic lymphocytic leukemia; MPAL: mixed phenotype acute leukemia. χ²: chi-square statistic; df: degrees of freedom. p-values derived from the chi-square test. A p-value of < 0.05 was considered statistically significant.

Clinical features	AML (n = 37)	B-ALL (n = 37)	T-ALL (n = 13)	CML-CP (n = 42)	CLL (n = 19)	MPAL (n = 3)	χ²	df	p-value
Fever	36 (97.3%)	34 (91.9%)	10 (76.9%)	7 (16.7%)	10 (52.6%)	3 (100%)	29.1	4	< 0.001
Bleeding manifestations	24 (64.9%)	24 (64.9%)	9 (69.2%)	6 (14.3%)	3 (15.8%)	3 (100%)	68.9	4	< 0.001
Lymphadenopathy	10 (27.0%)	4 (10.8%)	3 (23.1%)	9 (21.4%)	19 (100%)	2 (66.7%)	47.2	4	< 0.001
Splenomegaly	20 (54.1%)	23 (62.2%)	9 (69.2%)	42 (100%)	17 (89.5%)	3 (100%)	43.1	4	< 0.001
Hepatomegaly	20 (54.1%)	22 (59.5%)	7 (53.8%)	31 (73.8%)	12 (63.2%)	3 (100%)	4.64	4	0.326
Sternal tenderness	7 (18.9%)	10 (27.0%)	4 (30.8%)	2 (4.8%)	2 (10.5%)	0 (0%)	8.77	4	0.067

Fever was more commonly observed in acute leukemia subtypes, in contrast to chronic variants. Bleeding manifestations were also more prevalent in acute variants. Lymphadenopathy was universal in CLL cases, and splenomegaly was present in all CML-CP cases. All three MPAL cases presented with both fever and bleeding.

The mean hemoglobin was lowest in MPAL (6.7 ± 0.6 g/dL) and highest in CLL (10.1 ± 1.4 g/dL). AML and B-ALL presented with severe anemia (35.1% each), which was absent in CLL. Median TLC was highest in CML-CP (153,500/mm³), with hyperleukocytosis in 78.6% of cases. Severe thrombocytopenia was observed in almost all cases of acute leukemia, was rare in CML-CP (4.8% of cases), and was absent in CLL. Bone marrow blast percentage was markedly elevated in acute leukemias compared to chronic variants (Table 3).

**Table 3:** Hematological parameters across hematological malignancy subtypes (n = 151) AML: acute myeloid leukemia; B-ALL: B-cell acute lymphoblastic leukemia; T-ALL: T-cell acute lymphoblastic leukemia; CML-CP: chronic myeloid leukemia, chronic phase; CLL: chronic lymphocytic leukemia; MPAL: mixed phenotype acute leukemia; Hb: hemoglobin; WBC: white blood cell count; BMA: bone marrow aspiration; SD: standard deviation; F: Welch's F statistic; H: Kruskal-Wallis H statistic. Data for hemoglobin and bone marrow blast percentage are presented as mean ± SD; data for WBC and platelet counts are presented as median (range). p-values for hemoglobin and bone marrow blast percentage derived from Welch's one-way ANOVA; p-values for WBC count and platelet count derived from the Kruskal-Wallis test. † Denotes sub-group analysis within the respective parameter; it is not included in the overall statistical comparison. N/A: Test statistic and p-value not applicable for subgroup frequency analyses. A p-value of < 0.05 was considered statistically significant.

Hematological parameter	AML (n = 37)	B-ALL (n = 37)	T-ALL (n = 13)	CML-CP (n = 42)	CLL (n = 19)	MPAL (n = 3)	Test statistic	p-value
Hb (g/dL) mean ± SD	7.0 ± 1.3	7.1 ± 1.2	7.0 ± 0.8	8.5 ± 1.4	10.1 ± 1.4	6.7 ± 0.6	F = 23.4	< 0.001
— Severe anemia (Hb <7 g/dL)†	13 (35.1%)	13 (35.1%)	2 (15.4%)	3 (7.1%)	0 (0%)	1 (33.3%)	N/A	N/A
WBC (/mm³) median (range)	19200 (5800–88400)	17200 (4600–42000)	12800 (9800–44000)	153500 (28600–340000)	69000 (35000–145000)	45000 (12000–125000)	H = 148.2	< 0.001
— Hyperleukocytosis (>100000/mm³)†	0 (0%)	0 (0%)	0 (0%)	33 (78.6%)	5 (26.3%)	1 (33.3%)	N/A	N/A
Platelets (/mm³) median (range)	22000 (11000–145000)	19000 (12000–125000)	26000 (12000–54000)	312500 (12000–680000)	175000 (95000–240000)	22000 (15000–45000)	H = 182.5	< 0.001
— Severe thrombocytopenia (<50000/mm³)†	35 (94.6%)	34 (91.9%)	12 (92.3%)	2 (4.8%)	0 (0%)	3 (100%)	N/A	N/A
Blast% on BMA mean ± SD	62.7 ± 16.7	65.9 ± 14.5	60.7 ± 11.6	4.5 ± 4.4	1.1 ± 1.3	65.7 ± 18.9	F = 336.1	< 0.001
— High blast count (>50%)†	30 (81.1%)	31 (83.8%)	11 (84.6%)	0 (0%)	0 (0%)	2 (66.7%)	N/A	N/A

**Figure 1:**
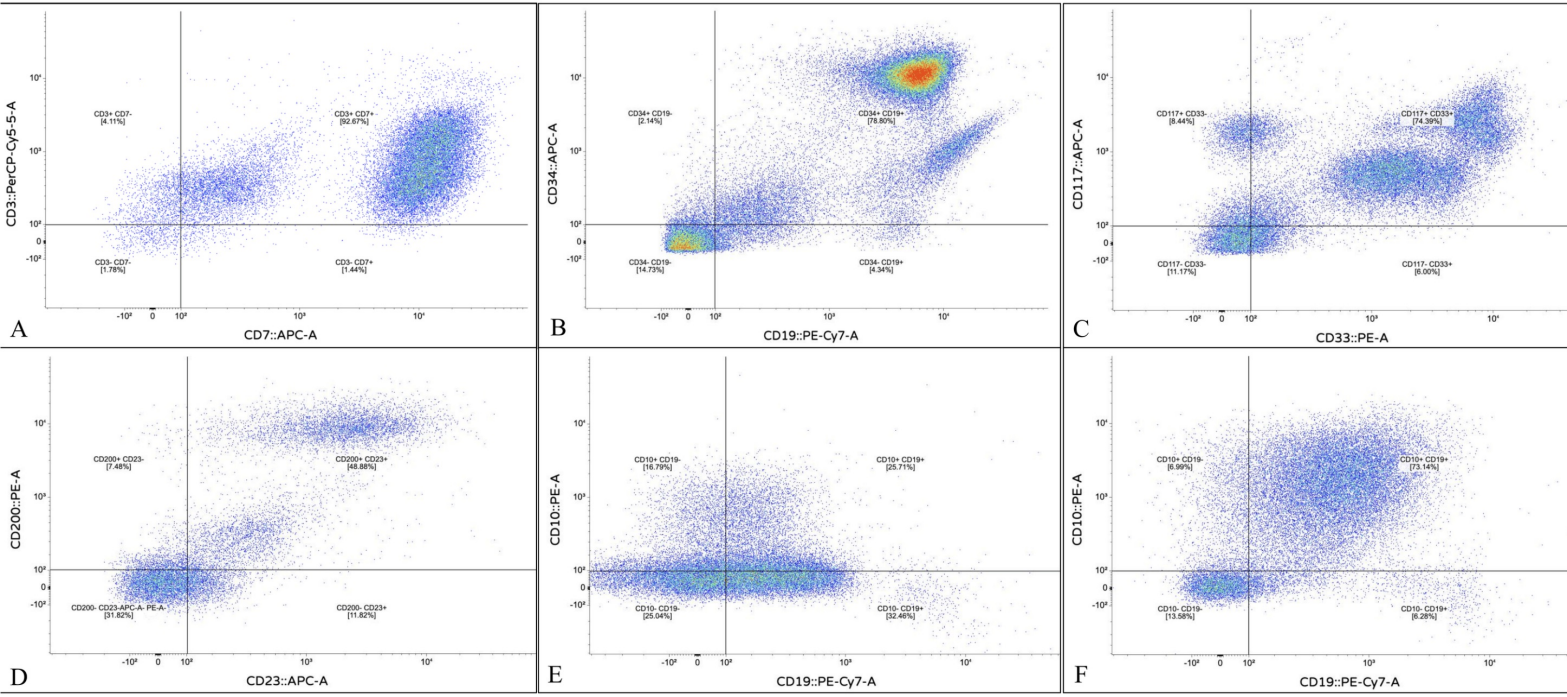
Representative flow cytometry dot plots demonstrating immunophenotypic marker co-expression patterns across hematological malignancy subtypes (A) CD3 vs. CD7 co-expression showing a dominant CD3+CD7+ population (92.67%), representative of T-cell acute lymphoblastic leukemia (T-ALL); (B) CD34 vs. CD19 co-expression showing a dominant CD34+CD19+ population (78.80%), representative of a CD34-positive B-cell acute lymphoblastic leukemia (B-ALL) case; (C) CD117 vs. CD33 co-expression showing a dominant CD117+CD33+ population (74.39%), representative of acute myeloid leukemia (AML); (D) CD200 vs. CD23 co-expression showing a dominant CD200+CD23+ population (48.88%), representative of chronic lymphocytic leukemia (CLL); (E) CD10 vs. CD19 showing a CD10−CD19+ dominant pattern (32.46%), representative of a CD10-negative B-ALL case (pro-B ALL subtype); (F) CD10 vs. CD19 showing a dominant CD10+CD19+ population (73.14%), representative of a CD10-positive B-ALL case (common ALL subtype).

The predominant myeloid markers in AML were CD33 (89.2%), CD13 (81.1%), cMPO (75.7%), CD117 (67.6%), and HLA-DR (67.6%). CD19 was universally expressed in B-ALL cases, followed by TdT (94.6%), CD79a (91.9%), CD10 (86.5%), and CD34 (18.9%). CD20 expression was low at 8.1% of cases. Aberrant myeloid marker expression was observed in four B-ALL cases (10.8%), all in the absence of cMPO expression. The pattern varied across cases; two cases expressed CD13 alone, one expressed CD33 alone, and one co-expressed both CD13 and CD33. All T-ALL cases expressed cCD3, CD5, and CD7 (100% each), followed by CD34 and TdT, each in 92.3% of cases. CLL expressed CD19, CD5, and CD200 in all cases (100% each), followed by CD23 (n = 17; 89.5%) and CD79a (n = 15; 78.9%) (Figure 1).

The MPAL cases were classified by immunophenotyping; two were B/myeloid MPAL, and one was T/myeloid MPAL. The B/myeloid cases co-expressed B-lineage markers (CD19, cytoplasmic CD79a (cCD79a); and CD19, CD10, and HLA-DR, respectively) alongside myeloid markers (CD13, CD33, and cMPO; and CD13, CD117, and cMPO, respectively). The T/myeloid case co-expressed T-lineage markers (cCD3 and CD7) with myeloid markers (CD13, CD33, and cMPO). Lineage assignment was made in accordance with WHO-HAEM5 and ICC scoring criteria [7-10].

CD34-positive AML (n = 23) showed a significantly higher WBC count (54,000 ± 35,014 vs. 28,600 ± 18,200/mm³; p = 0.006), a lower hemoglobin level (6.2 ± 1.9 vs. 8.4 ± 1.4 g/dL; p < 0.001), a lower platelet count (24,500 ± 20,999 vs. 45,000 ± 27,600/mm³; p = 0.024), and a higher bone marrow blast percentage (74.8 ± 15.9% vs. 52.6 ± 12.5%; p < 0.001) compared to CD34-negative cases (n = 14). In B-ALL, CD10-negative cases (n = 5) had a significantly higher WBC count (median 60,000 vs. 15,500/mm³; p < 0.001), a higher bone marrow blast percentage (median 81.0% vs. 64.0%; p = 0.008), and a lower platelet count (median 32,000 vs. 46,000/mm³; p = 0.048) compared to CD10-positive cases (n = 32). Hemoglobin levels did not differ significantly between groups (p = 0.724). Given the small number of CD10-negative cases (n = 5), these findings should be regarded as exploratory. The antigen expression and disease severity parameters are summarized in Tables 4, 5.

**Table 4:** Association of CD34 expression with disease severity parameters in AML (n = 37) AML: acute myeloid leukemia; WBC: white blood cell count; SD: standard deviation; t: Welch's independent samples t-statistic. Data presented as mean ± SD. p-values derived from Welch's independent samples t-test (unequal variances assumed). A p-value of < 0.05 was considered statistically significant.

Parameter	CD34-positive (n=23)	CD34-negative (n=14)	Welch's t	p-value
WBC (/mm³)	54,000 ± 35,014	28,600 ± 18,200	2.89	0.006
Hemoglobin (g/dL)	6.2 ± 1.9	8.4 ± 1.4	−4.07	<0.001
Platelets (/mm³)	24,500 ± 20,999	45,000 ± 27,600	−2.39	0.024
BM Blast %	74.8 ± 15.9	52.6 ± 12.5	4.72	<0.001

**Table 5:** Association of CD10 expression with disease severity parameters in B-ALL (n = 37) B-ALL: B-cell acute lymphoblastic leukemia; WBC: white blood cell count; IQR: interquartile range; U: Mann-Whitney U statistic. Data presented as median (IQR). p-values derived from the Mann-Whitney U test, used given the small sample size of the CD10-negative subgroup (n = 5). A p-value of < 0.05 was considered statistically significant.

Parameter	CD10-positive (n=32)	CD10-negative (n=5)	U-Statistic	p-value
WBC (/mm³)	15500 (7500–28000)	60000 (35000–85000)	15.5	<0.001
Hemoglobin (g/dL)	7.2 (7.0–7.4)	6.8 (6.0–7.8)	74	0.724
Platelets (/mm³)	46000 (38000–55000)	32000 (25000–40000)	34.5	0.048
BM Blast %	64.0 (55.0–72.0)	81.0 (75.0–86.0)	22	0.008

## Discussion

The present study evaluates the clinico-hematological and immunophenotypic profile of 151 newly diagnosed leukemia cases from a tertiary care center in Jharkhand. This is one of the few such comprehensive analyses from the eastern part of India. Jharkhand currently lacks a government-maintained population-based cancer registry, thus making a tertiary care hospital study essential for establishing a regional baseline dataset [6].

Spectrum and distribution of leukemia

The majority of cases in the study population were acute leukemias, which is consistent with other tertiary care-based studies by Shuchismita et al. [11] from Bihar, Biswas et al. [16] from West Bengal, and Kumar et al. [17] from Central India. The rapid symptomatic onset of acute leukemias compels earlier healthcare seeking, accounting for their predominance in a hospital-based study [4]. CML-CP was the most frequent diagnosis (27.8%), and all 42 cases were confirmed BCR-ABL1 positive by RT-PCR. Such disproportionately high CML proportions compared to Western literature are a common finding in other South Asian studies [3,18]. This may possibly reflect occupational and environmental exposures prevalent in the largely agrarian and industrial population of Jharkhand, though such exposures were not directly assessed in this study, and this interpretation remains speculative.

Among the acute leukemias, equal numbers of AML and B-ALL cases were diagnosed. This is in contrast with Western population data, where acute lymphoblastic leukemia (ALL) predominates across all age groups [4]. This lower ALL proportion may be attributed to referral bias of adult patients to tertiary centers, lower pediatric healthcare access in the region, and underrepresentation of patients from lower socioeconomic strata, who may present late or not at all.

Demographic profile

Male predominance was observed across all subtypes (74.2%; M:F = 2.9:1), which is consistent with other Indian studies by Shuchismita et al. [11], Kumar et al. [17], and Arooj et al. [18], which report ratios ranging from 1.5:1 to over 3:1. Socioeconomic factors, occupational exposures, and cultural patterns of healthcare access are likely responsible for this disparity.

The mean age at diagnosis was lower in this study compared to Western data, but it is consistent with other studies conducted in India [4,11]. India's younger demographic profile and higher mortality in elderly patients before diagnosis in resource-limited healthcare centers likely account for this difference.

Clinical presentation

Fever was the most common clinical presentation, especially in acute variants (AML: 97%, ALL: 88%, MPAL: 100%), which aligns with Shuchismita et al. [11] and Munirathinam et al. [19]. The cause of fever is attributed to neutropenia-induced immunosuppression and infection, cytokine release from blast cells, and a tumor-induced inflammatory response. Bleeding manifestations were predominantly observed in acute leukemia subtypes (p < 0.001), closely correlating with the degree of thrombocytopenia, which is consistent with the findings of Munirathinam et al. [19] and Şenol et al. [20]. Lymphadenopathy was found in all CLL cases and was frequent in ALL; however, it was infrequent in AML cases. This is due to the bone marrow-predominant nature of myeloid disease and the higher propensity for extranodal involvement in the lymphoid variants of leukemia [21]. Splenomegaly was seen in all CML-CP cases, consistent with characteristic extramedullary hematopoiesis [18].

Hematological parameters

Moderate to severe anemia was almost universal in acute leukemias, consistent with the findings of Shuchismita et al. [11] and Zaidi et al. [22]. Lower hemoglobin levels at presentation compared to Western population data can be attributed to late presentation due to a lack of awareness and specialized healthcare in the region. The TLC showed wide variation across the cohort, reflecting the heterogeneous mix of subtypes studied. Hyperleukocytosis was found in 78.6% of CML cases, consistent with the study by Arooj et al. [18], while acute leukemias showed leukopenia or moderate leukocytosis, consistent with Zaidi et al. [22] and Wiraatmadja et al. [23]. Severe thrombocytopenia was almost universal in acute leukemias and is consistent with the findings of Şenol et al. [20] and Wiraatmadja et al. [23]. The markedly elevated blast percentage in acute variants compared to chronic subtypes was statistically significant (p < 0.001), similar to findings reported by Zaidi et al. [22] and Wiraatmadja et al. [23]. Additionally, the higher prevalence of nutritional deficiencies, parasitic infestations, and hemoglobinopathies in this population may contribute to the lower baseline hemoglobin at presentation compared to Western data, compounding the anemia of malignancy.

Immunophenotypic profile

Morphological assessment on PBS and BMA forms the initial diagnostic basis; however, only up to 80% of cases can be correctly diagnosed and classified based on morphology alone, especially for acute subtypes [24]. Flow cytometry adds diagnostic precision, consistent with Gupta et al. [24], who reported above 90% accuracy with immunophenotyping. Various national and international consensus guidelines recommend flow cytometry-based immunophenotyping for subtyping and lineage classification [7,8,14].

CD34 positivity in AML correlated significantly with higher TLC, lower hemoglobin and platelet counts, and higher blast percentage (all p < 0.05 by Welch's t-test), suggesting a more proliferative and primitive phenotype [10,25]. CD10-negative B-ALL was associated with higher WBC count (p < 0.001), higher blast percentage (p = 0.008), and lower platelet count (p = 0.048) by the Mann-Whitney U test, consistent with the biological profile of high-risk pro-B-ALL [7,8]. However, given the small number of CD10-negative cases (n = 5), these findings should be regarded as exploratory. Adequately powered studies are needed to confirm these observations. Aberrant myeloid markers were observed in four B-ALL cases, with CD13 and CD33 expressed in the absence of cMPO, a recognized finding that does not alter lineage assignment but may be relevant for potential therapeutic consideration [8,10].

Limitations

First, this was a single-center, hospital-based study from a tertiary referral center; therefore, findings are subject to referral bias and may not represent the true burden of leukemia in the region. Second, the absence of cytogenetic and molecular analyses due to infrastructural constraints limited definitive WHO-HAEM5-based genetic subclassification, limiting the diagnostic precision to morphological and immunophenotypic categorization for most entities. Third, French-American-British (FAB) subtyping was not performed, limiting intra-subtype granularity. Finally, as a cross-sectional descriptive study, treatment and follow-up data were unavailable; survival and remission analysis were, therefore, beyond the scope of this study.

## Conclusions

The findings of this study provide a comprehensive baseline of the clinico-hematological and immunophenotypic profile of leukemias in Jharkhand. These findings reflect the patterns reported by studies from other eastern Indian states, underscoring a shared regional healthcare challenge. These data highlight the need for a state-level population-based cancer registry in Jharkhand and for strengthening appropriate diagnostic and treatment infrastructure, and may facilitate evidence-based policymaking.
